# Exploring the Link Between Intraocular Pressure and Central Corneal Thickness, as Well as the Diurnal Variation of Intraocular Pressure, in the Elderly Population of North Karnataka

**DOI:** 10.7759/cureus.64022

**Published:** 2024-07-07

**Authors:** Rekha Mudhol, Lingamaneni Sneha

**Affiliations:** 1 Ophthalmology, Shri BM Patil Medical College Hospital and Research Centre, Bijapur Lingayat District Educational Association (Deemed to be University), Vijayapura, IND

**Keywords:** population-based study, diurnal variation, central corneal thickness, intraocular pressure, age

## Abstract

Purpose

The study aimed to understand the relationship between intraocular pressure (IOP) and central corneal thickness (CCT) in older adults living in North Karnataka and the diurnal variation of IOP in the elderly population.

Methods

This is a population-based cross-sectional study in which 84 eyes of 42 study participants aged over 50 years were examined. A complete ophthalmic examination was done for all subjects. IOP was measured with an iCare IC100 (Icare Finland Oy, Vantaa, Finland) rebound tonometer and CCT with an ultrasound pachymeter. Statistical significance was accepted as p<0.05.

Results

The mean age of the study participants was 64.42±6.917 years. The mean IOP and CCT at 1 pm were 12.4±2.576 and 509.24±25.379 in the right eye, and 12.45±2.319 and 511.05±24.464 in the left eye. Spearman’s correlation showed that IOP was positively correlated with CCT, with p<0.05. This showed that CCT has the most significant impact on IOP. In our study, the diurnal variation of IOP by paired samples t-test was also crucial in the elderly population, with p<0.05.

Conclusion

In this study, IOP was reduced as the CCT was reduced, and vice versa, i.e., IOP was positively correlated with CCT. The diurnal variation of IOP in older people was the same as in the young and middle-aged population, i.e., the highest recording of IOP was in the morning, and it gradually reduced as the day passed.

## Introduction

Glaucoma is the leading cause of permanent blindness globally [1]. Even with modern and advanced diagnostic technologies, it is still difficult to diagnose the progression of glaucoma, and it is, therefore, vital to carefully monitor and treat glaucoma patients [2].

Since intraocular pressure (IOP) is the only modifiable risk factor for glaucoma, it is essential to understand and effectively treat it [2-11]. Therefore, it is imperative to know IOP and the factors that impact it [11]. IOP varies with age, gender, central corneal thickness (CCT), body mass index, systemic hypertension, diabetes, iris color, alcohol, and cigarette. Also, knowing the daily fluctuations of IOP will help make treatment adjustments [8,11-13].

Thinner corneas underestimate the IOP, while the thicker corneas overestimate [9,13,14]. As CCT affects IOP, it is essential to estimate it in glaucoma patients [7,9].

Values of IOP and CCT depend on the consistency and accuracy of their measurements [10]. The Goldmann applanation tonometer gives the most accurate IOP values, and the ultrasonic pachymeter gives accurate CCT values [10,14]. The rebound tonometer is as precise as the Goldmann applanation tonometer, more reliable than the non-contact tonometer, and portable [10]. Hence, the rebound tonometer (iCare IC100; Icare Finland Oy, Vantaa, Finland) and ultrasonic pachymeter were used in this study. It has been proposed that IOP variations and absolute IOP levels are crucial for advancing disease. There are conflicting and divergent views regarding the reliability of daily pressure profiles [15].

Many studies have determined the relationship between IOP and CCT, but the results are controversial [7]. Only a small number of studies have addressed IOP in the older population. The current study was conducted to investigate the diurnal variation of IOP in older persons and to comprehend the association between IOP and CCT in the elderly population of North Karnataka, in light of the contentious results.

## Materials and methods

This study was carried out in a single day on 42 individuals aged 50 and above from North Karnataka who sought medical care at the Ophthalmology Outpatient Department of Shri BM Patil Medical College Hospital and Research Centre, Vijayapura, India. Participation was optional and authorized by the Institute’s Ethics Committee with approval number BLDE (DU)/IEC/1061-F/2023-24. The study did not include individuals with ocular disorders like glaucoma, mature and hyper-mature cataracts, history of any ocular surgery in the past, corneal pathology, or systemic diseases that hindered the measurement and assessment of IOP and CCT. 

IOP and CCT are compared using Spearman’s rank correlation coefficient (r). Wilcoxon signed-rank test (W-value) was used to study the diurnal variation of IOP.

A thorough medical history was obtained, and each participant got a comprehensive eye examination, including a visual acuity assessment and slit lamp examination.

Since the iCare rebound tonometer gives values as accurate as the Goldmann tonometer and as it is portable, the IOP of both eyes was assessed using a rebound tonometer (iCare IC100) at four specific time points during the day: 7 am, 1 pm, 6 pm, and 12 am in a sitting position, always after resting for 15 minutes [10,12]. The CCT of both eyes was measured at 1 pm in a sitting position using an ultrasonic pachymeter. 

Quantitative data analysis

The obtained data were documented in a Microsoft Excel (Microsoft® Corp., Redmond, WA, USA) spreadsheet and analyzed using IBM SPSS Statistics for Windows, Version 20 (Released 2011; IBM Corp., Armonk, NY, USA). The outcomes are presented as averages, variances, totals, and proportions, and visualized using diagrams. A paired samples t-test assessed the diurnal variation of IOP in the right and left eye over a day. Comparing categorical data between groups was done using the Chi-square or Fisher’s exact test. A p-value below 0.05 was deemed to be statistically significant. The statistical tests conducted were two-tailed [11].

## Results

This study included 84 eyes of 42 individuals over 50 residing in North Karnataka. Of the 42 individuals, 66.7% were females, and 33.3% were males. As can be observed in Table 1, the average age of the study participants was 64.42±6.917. 

**Table 1:** Demographic analysis of the study population

Age distribution						
Participants	Age range		Average age			Standard deviation
42	50 to 78 years		64.62			6.917
Gender distribution						
Gender		Count		Percentage	Cumulative percentage	
Female		28		66.7%	66.7%	
Male		14		33.3%	33.3%	
Total no. of participants		42		100%	100%	

Table 2 summarizes the average IOP and CCT measured in the study. The highest average IOP was recorded at 7 am, with measurements of 13.12±2.189 mmHg for the right eye and 12.9±2.151 mmHg for the left eye. The minimum average IOP was observed at midnight, with measurements of 11.5±1.811 mmHg for the right eye and 11.48±1.77 mmHg for the left eye, indicating the daily fluctuation in IOP. The mean CCT was 509.24±25.379 µm for the right eye and 511.05±24.464 µm for the left eye, with CCT values ranging from 470 to 580 µm. The paired sample t-test conducted on the daily fluctuation in IOP in both eyes produced statistically significant findings (p<0.05), as seen in Table 3.

**Table 2:** Mean and standard deviation of IOP and CCT of the right and the left eye in the study population All IOP values in mmHg and CCT values in micrometer (µm) IOP: Intraocular pressure; CCT: Central corneal thickness; IOPR: Intraocular pressure of the right eye; IOPL: Intraocular pressure of the left eye; CCTR: Central corneal thickness of the right eye; CCTL: Central corneal thickness of the left eye; N: Number of participants

Parameter	N	Minimum	Maximum	Mean	Std. deviation
IOPR - 7 am	42	9	18	13.12	2.189
IOPR - 1 pm	42	8	19	12.4	2.576
IOPR - 6 pm	42	8	17	11.9	2.081
IOPR - 12 am	42	8	16	11.5	1.811
IOPL - 7 am	42	8	18	12.9	2.151
IOPL - 1 pm	42	9	19	12.45	2.319
IOPL - 6 pm	42	8	18	11.62	2.358
IOPL - 12 am	42	9	16	11.48	1.77
CCTR	42	470	580	509.24	25.379
CCTL	42	474	572	511.05	24.464

**Table 3:** Diurnal variation of IOP in the study population IOP: Intraocular pressure; IOPR: Intraocular pressure of the right eye; IOPL: Intraocular pressure of the left eye * A p-value below 0.05 was deemed to be statistically significant

Measure 1	Measure 2	W-value	p-value
Paired samples t-test (left eye)			
IOPL - 7 am	IOPL - 1 pm	368.5	0.047*
IOPL - 7 am	IOPL - 6 pm	523	<0.001*
IOPL - 7 am	IOPL - 12 am	545	<0.001*
Paired samples t-test (right eye)			
IOPR - 7 am	IOPR - 1 pm	382	0.002*
IOPR - 7 am	IOPR - 6 pm	699.5	<0.001*
IOPR - 7 am	IOPR - 12 am	719	<0.001*

**Figure 1:**
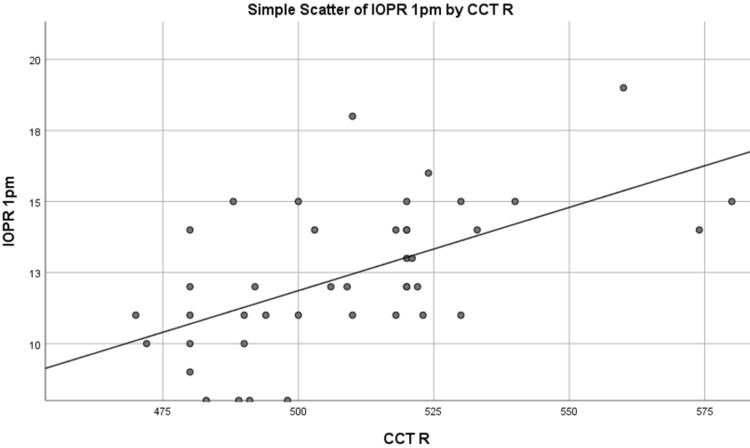
Graph representation of the correlation between IOPR and CCTR at 1 pm IOP values in mmHg and CCT values in micrometers IOP: Intraocular pressure; CCT: Central corneal thickness; IOPR: Intraocular pressure of the right eye; CCTR: Central corneal thickness of the right eye

**Figure 2:**
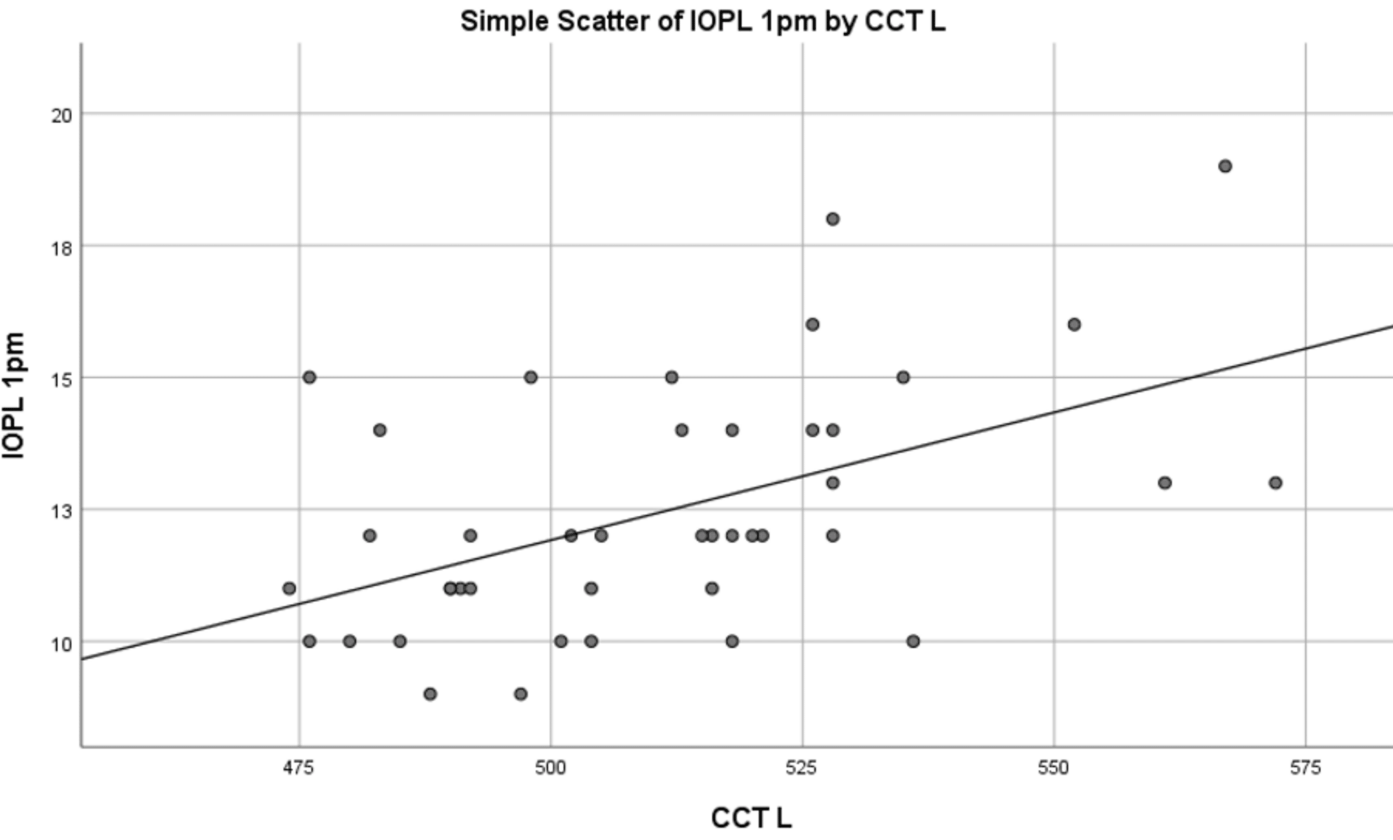
Graph representation of the correlation between IOPL and CCTL at 1 pm IOP values in mmHg and CCT values in micrometers IOP: Intraocular pressure; CCT: Central corneal thickness; IOPL: Intraocular pressure of the left eye; CCTL: Central corneal thickness of the left eye

IOP measured at 1 pm was correlated with CCT measured at 1 pm using Spearman’s rank correlation coefficient and was significant in both right and left eyes, as shown in Table 4, and is represented by graphs shown in Figures 1-2. A direct relationship was observed between IOP and CCT. This proves that CCT impacts IOP and is an important parameter to be checked in glaucoma patients when IOP is measured.

**Table 4:** Relation between IOP and CCT at 1 pm IOP: Intraocular pressure; CCT: Central corneal thickness; IOPR: Intraocular pressure of the right eye; IOPL: Intraocular pressure of the left eye; CCTR: Central corneal thickness of the right eye; CCTL: Central corneal thickness of the left eye * A p-value below 0.05 was deemed to be statistically significant (highly significant correlation between the two variables (p<0.001), indicating a strong association)

		IOPR - 1 pm	CCTR
IOPR - 1 pm	Correlation coefficient	1.00	0.585
	Significance (2-tailed)	<0.001*	<0.001*
	Number of participants	42	42
CCTR	Correlation coefficient	0.585	1.00
	Significance (2-tailed)	<0.001*	<0.001*
	Number of participants	42	42
		IOPL - 1 pm	CCTL
IOPL - 1 pm	Correlation coefficient	1.000	0.495
	Significance (2-tailed)	<0.001*	<0.001*
	Number of participants	42	42
CCTL	Correlation coefficient	0.495	1.00
	Significance (2-tailed)	<0.001*	<0.001*
	Number of participants	42	42

## Discussion

This population-based study in North Karnataka investigated the distribution and fluctuation of IOP throughout the day in older people and its relationship with CCT. Given that glaucoma, a leading cause of irreversible blindness, has IOP as a critical modifiable risk factor [1-11], understanding the IOP distribution within this population is crucial for identifying potential abnormalities [8]. Furthermore, as CCT can influence IOP measurements, its assessment is vital in glaucoma patients [9,11,14].

Previous research has shown conflicting results on the connection between IOP and CCT [7]. Nonetheless, our results align with the research carried out by Alberta et al., where they showed a positive association between IOP and CCT of the right eye (correlation coefficient r=0.355, p=0.000) and the left eye (correlation coefficient r=0.381, p=0.000) [9]. 

Similarly, Irgat and Yildirim demonstrated a noteworthy and statistically significant positive correlation between IOP and CCT (r=0.137, p<0.001) [11].

Alkhodari found a moderate correlation between IOP and CCT in the right eye (r=0.358, p=0.000) and the left eye (r=0.324, p=0.000), similar to our study [16]. In a survey by Nejabat et al., there was also a direct relationship between CCT and IOP, with a significant p-value of 0.000 [17].

​Additionally, our study confirms the diurnal variation of IOP, consistent with earlier research [12]. The usual range of IOP is 10 to 21 mmHg. At one point in the day, IOP can be within the normal range (normotension), but there can be ocular hypertension at certain times of the day [12]. Hence, studying diurnal variation is essential. 

In our study, IOP was within the normal range, although there was a variation in IOP as the day passed. Like earlier research, our study did not find a statistically significant difference between the IOP of the two eyes [12]. Kim et al., similar to our research, found that non-glaucomatous patients had comparatively less frequent IOP asymmetry between bilateral eyes [18].

We observed the highest IOP measurements in the morning (13.12±2.189 mmHg in the right eye and 12.9±2.151 mmHg in the left eye), with levels decreasing as the day progresses (11.5±1.811 mmHg in the right eye and 11.48±1.77 mmHg in the left eye) in this elderly population, which is consistent with the study done by Bharathi and Bhuvaneswari [19] and Rana et al. [20], who showed high IOP readings in the early morning hours.

The main strength of this study is that all measurements for the 42 participants were taken on the same day, ensuring consistency. However, the small sample size is a limitation. Future research with larger sample sizes is necessary to explore further the relationship between IOP and CCT. Understanding this relationship is critical, as IOP is the only modifiable risk factor in the treatment of glaucoma.

## Conclusions

This study establishes a direct relationship between IOP and CCT. Notwithstanding this correlation, IOP and CCT measurements were within the normal range. The influence of CCT on IOP was substantial. Our research also revealed that the daily fluctuations in IOP in older individuals are similar to those observed in younger and middle-aged groups. The highest IOP measurements are often observed in the morning, followed by a progressive reduction during the day. Further research with a bigger sample size is necessary to validate these findings and improve our comprehension of the correlation between IOP and CCT in managing glaucoma.
